# A High-Throughput Gene Disruption Methodology for the Entomopathogenic Fungus *Metarhizium robertsii*

**DOI:** 10.1371/journal.pone.0107657

**Published:** 2014-09-15

**Authors:** Chuan Xu, Xing Zhang, Ying Qian, Xiaoxuan Chen, Ran Liu, Guohong Zeng, Hong Zhao, Weiguo Fang

**Affiliations:** Institute of Microbiology, College of Life Sciences, Zhejiang University, Hangzhou, Zhejiang, China; California Department of Public Health, United States of America

## Abstract

Systematic gene disruption is a direct way to interrogate a fungal genome to functionally characterize the full suite of genes involved in various biological processes. *Metarhizium robertsii* is extraordinarily versatile, and it is a pathogen of arthropods, a saprophyte and a beneficial colonizer of rhizospheres. Thus, *M. robertsii* can be used as a representative to simultaneously study several major lifestyles that are not shared by the “model” fungi *Saccharomyces cerevisiae* and *Neurospora crassa*; a systematic genetic analysis of *M. robertsii* will benefit studies in other fungi. In order to systematically disrupt genes in *M. robertsii*, we developed a high-throughput gene disruption methodology, which includes two technologies. One is the modified OSCAR-based, high-throughput construction of gene disruption plasmids. This technology involves two donor plasmids (pA-Bar-OSCAR with the herbicide resistance genes *Bar* and pA-Sur-OSCAR with another herbicide resistance gene *Sur*) and a recipient binary plasmid pPK2-OSCAR-GFP that was produced by replacing the *Bar* cassette in pPK2-bar-GFP with a *ccdB* cassette and recombination recognition sites. Using this technology, a gene disruption plasmid can be constructed in one cloning step in two days. The other is a highly efficient gene disruption technology based on homologous recombination using a *Ku70* deletion mutant (*ΔMrKu70*) as the recipient strain. The deletion of *MrKu70*, a gene encoding a key component involved in nonhomologous end-joining DNA repair in fungi, dramatically increases the gene disruption efficiency. The frequency of disrupting the conidiation-associated gene *Cag8* in *ΔMrKu70* was 93% compared to 7% in the wild-type strain. Since *ΔMrKu70* is not different from the wild-type strain in development, pathogenicity and tolerance to various abiotic stresses, it can be used as a recipient strain for a systematic gene disruption project to characterize the whole suite of genes involved in the biological processes of *M. robertsii*.

## Introduction

The genus *Metarhizium* includes the best-studied entomopathogenic fungi at the molecular and biochemical levels. They have a worldwide distribution from the arctic to the tropics and colonize an impressive array of environments including forests, savannahs, swamps, coastal zones and desserts [1]. The representative species of the genus, *Metarhizium robertsii*, is extraordinarily versatile. This versatility derives from the fact that it is a pathogen of arthropods [1] and a saprophyte [2], and some isolates such as ARSEF2575 (used in this study) are the beneficial colonizers of rhizospheres (the layer of soil influenced by root metabolism) [3], [4]. Consequently, *M. robertsii* experiences several distinctive sets of selective pressures [5], [6], allowing the function of a gene to be assessed during the three major fungal lifestyles including parasitism, saprophytism and symbiotism. This is not matched by the “model” fungi *Saccharomyces cerevisiae* and *Neurospora crassa* [7].

Systematic gene disruption is a direct way to interrogate a fungal genome to functionally characterize the full suite of genes involved in various developmental processes. The completion of a systematic gene disruption project for a fungus is dependent on two important technologies. The first is a highly efficient gene disruption technology based on homologous recombination, and this technology is particularly important for filamentous fungi because they usually have a very low homologous recombination frequency [8], [9]. This is because filamentous fungi preferentially use nonhomologous end-joining DNA repair (NHEJ) in double-strand break (DSB) repair. As a result, exogenous DNA can be randomly integrated in the genome, even if it carries a long stretch of homologous sequence [8]. NHEJ is mediated by the DNA-dependent protein kinase catalytic subunit (DNA-PKcs), the Ku70-Ku80 heterodimer, and the DNA ligase IV-Xrcc4 complex. Deletion of the genes encoding Ku proteins impairs NHEJ (i.e., decreases the rate of non-homologous recombination), and thus significantly increases the rate of homologous recombination in the filamentous fungi [10]. Furthermore, the deletion of *Ku* genes does not alter their growth, development and infection [8], [9], [11], [12]. Therefore, mutants containing *Ku* deletions are used as recipient strains for systematic gene disruption in *N. crassa* and also for functionally characterizing genes involved in various biological processes in other fungi [8], [9], [11], [12]. The second technology is high-throughput approaches to generate gene disruption constructs. Such approaches include double-joint PCR fusion in the PCR-based, split-marker deletion method [13] and OSCAR-based (One Step Construction of *Agrobacterium* Recombination ready plasmids) construction of gene disruption plasmids [14]. Protoplast preparation and transformation are needed for the PCR-based split-marker deletion method, and *A. tumefaciens*-mediated fungal transformation is needed for the OSCAR-based technology.

The PCR-based, split-marker deletion method appears not to be suitable for *M. robertsii* mainly because the efficiency of protoplast-mediated *M. robertsii* transformation is very low [15]. In contrast, *A. tumefaciens*-mediated *M. robertsii* transformation is highly efficient. The current strategy for constructing plasmids for *A. tumefaciens*-mediated gene disruption in *M. robertsii* requires at least two rounds of restriction enzyme digestion and ligation, which is labor intensive and thus not feasible for high-throughput gene deletion. In this study, we describe a modified OSCAR methodology that facilitates the fast and high-throughput generation of gene disruption plasmids for *M. robertsii*. Disruption of the *M. robertsii* gene *MrKu70* (homologous to Ku70 of *N. crassa*) showed that the efficiency of gene disruption based on homologous recombination in *ΔMrKu70* was much higher than that in the wild-type strain.

## Results

### Modified OSCAR-based technology for high-throughput construction of gene disruption plasmids

**Figure 1:**
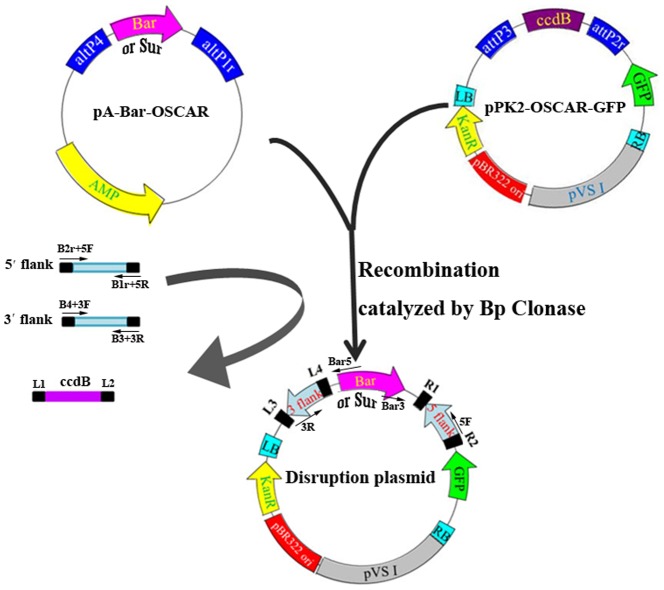
The modified OSCAR methodology for construction of gene disruption plasmids. The donor plasmids contain the cassette of the herbicide-resistance genes *Bar* or *Sur*. The recipient binary plasmid pPk2-OSCAR-GFP was used for construction of gene disruption plasmids in *E. coli*. Primers B2r+5F/B1r+5R and B4+3F/B3+3R were used for cloning 5′ and 3′ flanking sequences of the gene to be deleted, respectively. B2r, B1r, B4 and B3 are recognition sites of Bp Clonase for recombination, which were added to 5′ end of their respective primers. Primer 5F (or B2r+5F) and Bar3 (Sur3 when pA-Sur-OSCAR used) and Bar5 (Sur5 when pA-Sur-OSCAR used) and 3R (or B3+3R) were used to confirm the construction of the gene disruption plasmids.

In 2011, Paz et al. described an OSCAR technology that was a combination of PCR and the Gateway Technology and could generate a gene disruption plasmid with a single cloning step in just 2 days [14]. In this technology, the flanking region of the gene to be deleted are cloned by PCR and recombined into a donor plasmid (e.g., pA-Hyg-OSCAR) and a recipient binary plasmid (p-OSCAR) catalyzed by Bp Clonase (Life Technologies, USA), which generates the resultant gene disruption plasmid [14]. In order to use the OSCAR technology in *M. robertsii*, we modified both donor plasmids and the recipient binary plasmid. The hygromycin-resistance gene in pA-Hyg-OSCAR and nourseothricin-resistance gene in pA-NTC-OSCAR constructed by Paz et al. [14] are effective for *Verticillium dahlia* and many other fungi, but are not suitable for *M. robertsii*. Therefore, we replaced the HygR cassette in pA-Hyg-OSCAR with either of the two herbicide-resistance gene cassettes *Bar* and *Sur*, generating two new donor plasmids referred to as pA-Bar-OSCAR (Fig. 1) and pA-Sur-OSCAR (Fig. 1), respectively. To produce a recipient binary plasmid suitable for *M. robertsii*, the *Bar* cassette in the plasmid pPK2-bar-GFP [3] was replaced by the DNA fragment containing the recombination recognition sites (attp3 and attp2r) and the *ccdB* cassette from the plasmid p-OSCAR [14]. The new recipient binary plasmid is designated as pPK2-OSCAR-GFP (Fig. 1). The green fluorescent protein gene *egfp* in the plasmid can be used to aid in screening gene disruption mutants as described [16]. Taken together, we developed a modified OSCAR technology that contains two donor plasmids (pA-Bar-OSCAR and pA-Sur-OSCAR) and one recipient binary plasmid pPK2-OSCAR-GFP. The process of constructing gene disruption plasmids using these plasmids is outlined in Figure 1. Using this modified OSCAR technology, we successfully constructed many plasmids for disrupting *M. robertsii* genes. In this study, we describe the disruption of the conidiation-associated gene *Cag8* and the *M. robertsii MrKu70* gene, which is homologous to *Ku70* of *N. crassa* and is involved in NHEJ [8].

### Characterization of the *Ku70* gene in *M. robertsii*

**Figure 2:**
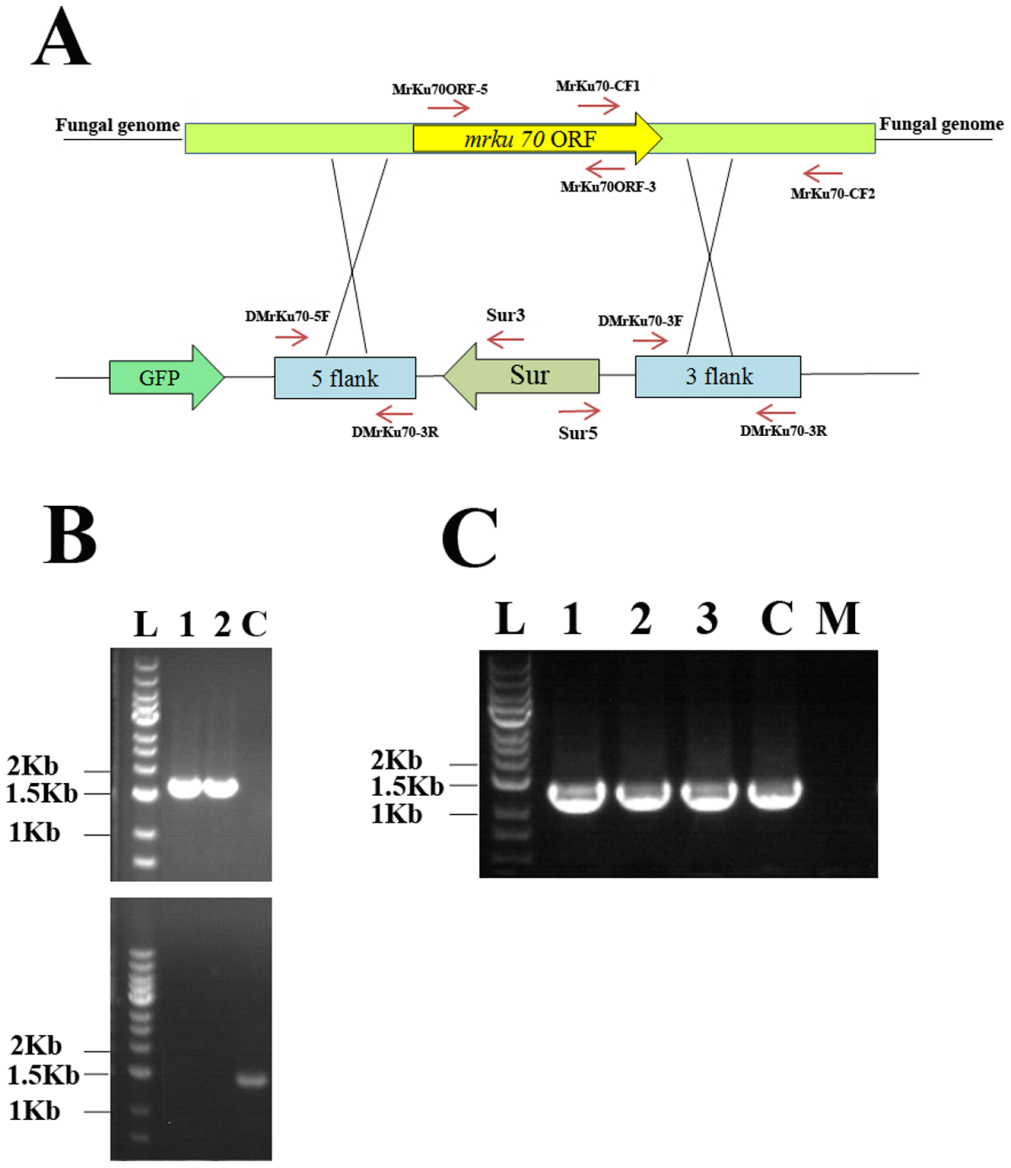
The disruption of *MrKu70* in *M. robertsii* using the herbicide-resistance gene *Sur* as the selection marker. (**A**) The disruption plasmid of *MrKu70* (bottom) and the relative position of *MrKu70* in the wild-type strain. (**B**) Confirmation of the disruption of *MrKu70* by PCR in the mutants with glufosinate ammonium resistance and without a GFP signal. 1 and 2 designate two different *ΔMrKu70* mutants, and C is the wild-type strain. Top panel: PCR conducted with the primers Sur5 and MrKu70CF2, PCR products can be obtained only from *MrKu70* disruption mutants; Bottom panel: PCR conducted using primers MrKu70CF1 and MrKu70CF2, and PCR products can be obtained in any strains other than *MrKu70* disruption mutants. (**C**) Confirmation of the complementation of *ΔMrKu70* by PCR using the primers MrKu70ORF-5 and MrKu70ORF-3 to amplify across the deleted region. 1 to 3: 3 different transformants with *ΔMrKu70* complemented; C: the wild-type strain; M: *ΔMrKu70*; L: DNA ladder (DL 10004) from Generay (Shanghai, China).

MrKu70 (Genbank accession number: XP_007817332) was identified by BLASTP using the Ku70 protein of *N. crassa* [8] as a query. MrKu70 consists of 646 amino acids containing a von Willebrand factor type A (vWFA)-like domain, a Ku-core domain (Ku70), and a DNA-binding domain (SAP domain). This domain structure is conserved in all characterized Ku70 orthologs of eukaryotic origin [8], [9], [11], [12]. MrKu70 exhibits 87, 82, 77, and 77% similarity to its orthologs found in *Trichoderma reesei* (ETS02014), *Trichoderma virens* (EHK20312), *N. crassa* (Q7SA95) and *Sordaria macrospora* (XP_003350038), respectively. We then constructed an *MrKu70* disruption plasmid using the modified OSCAR methodology with *Sur* as the selection marker, and disrupted *MrKu70* based on homologous recombination. Six disruption mutants (*ΔMrKu70*) were obtained from 200 transformants with chlorimuron ethyl resistance. *ΔMrKu70* was complemented by a genomic clone of *MrKu70.* The details of the disruption of *MrKu70* and the complementation of *ΔMrKu70* are described in Figure 2.

We tested the tolerance of *ΔMrKu70* conidia (two different mutants) to several abiotic stresses including UV-B, heat shock (45°C for 2 h), cold (15°C), high osmolarity (0.75 M KCl) and an oxidant (0.005% H_2_O_2_). No significant differences in tolerance to the abiotic stresses were observed between *ΔMrKu70*, the wild-type strain and the complemented *ΔMrKu70* (*P*>0.1) (data not shown). Bioassays with wax worm larvae showed the LT_50_ (the time taken to kill 50% of *G. mellonella* larvae) of the wild-type strain was 10.8±1.6 days, which was not significantly different from that of *ΔMrKu70* (11.0±1.9 days) and the complemented *ΔMrKu70* (11.1±1.2 days) (*P*>0.05). These results indicate that the disruption of *MrKu70* did not alter the pathogenicity of *M. robertsii* to this insect. *ΔMrKu70* also showed wild-type levels of germination in Sabouraud Dextrose Broth, growth and conidiation on Potato Dextrose Agar (PDA, Becton Dickinson and Company, France) (data not shown). In order to test the stability of the biological processes of two *ΔMrKu70*, the wild-type strain and the complemented *ΔMrKu70*, they were successively subcultured on PDA plates five times. Conidia from each subculture were collected and subjected to the same tests described above. No significant differences in the biological processes tested were found between subcultures of *ΔMrKu70*, the wild-type strain and the complemented *ΔMrKu70* (*P*>0.05). These data suggest that *ΔMrKu70* can be used as a recipient strain for gene disruption to functionally characterize genes involved in pathogenesis, development or tolerance to abiotic stresses in *M. robertsii*.

### Deletion of *MrKu70* increased gene disruption efficiency in *M. robertsii*

**Figure 3:**
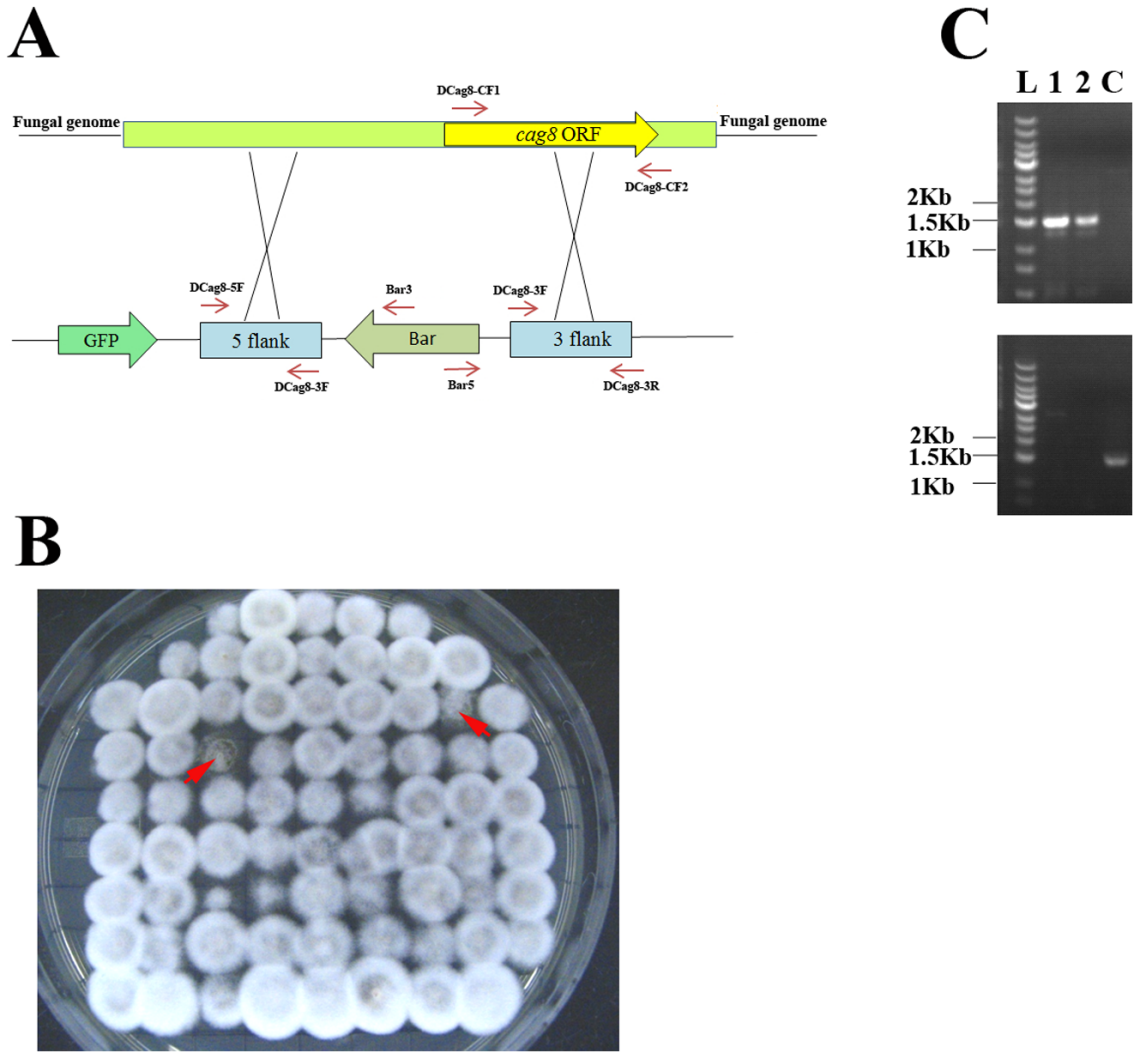
Disruption of *Cag8* in *ΔMrKu70* using the *bar* gene as the selection marker. (**A**) the *Cag8* disruption plasmid (pPK2-OSCAR-GFP-Cag8) and the relative position of *Cag8* in *ΔMrKu70*. (**B**) The representative 74 of 301 glufosinate ammonium-resistant transformants randomly selected from fungal transformation plates for *Cag8* disruption in *ΔMrKu70*. Arrows indicate colonies with conidiation, and the remaining 72 transformants show the fluffy phenotype without conidiation. (**C**) Two fluffy colonies were randomly selected from the transformation (B) (labeled as 1 and 2) for confirmation of *Cag8* disruption in *ΔMrKu70*. Top panel: PCR conducted with the primers Bar5 and Cag8CF2, PCR products can be obtained only from *ΔCag8:ΔMrKu70* mutants; Bottom panel: PCR conducted using primers Cag8CF1 and Cag8CF2, and PCR products can be obtained in any strain other than *ΔCag8:ΔMrKu70* mutants. L: DNA ladder (DL 10004) from Generay (Shanghai, China).

In order to test the gene disruption efficiency in *ΔMrKu70*, a well-defined *Cag8* gene in *M*. *robertsii* was selected as a target for disruption. The deletion of *Cag8* resulted in a fluffy phenotype without conidiation [17], and this visible phenotype change can allow us to readily evaluate the efficiency of gene disruption (Fig. 3B). The disruption plasmid for *Cag8* was generated using the modified OSCAR methodology with *Bar* as the selection marker, and the 5′ and 3′ flanking regions were 994 and 1,286 bp, respectively. Only 21 out of 325 glufosinate ammonium-resistant transformants (6.5%) from the wild-type strain were fluffy without conidia, while 281 out of 301 transformants (93.4%) from *ΔMrKu70* were fluffy without conidia (Fig. 3B). PCR confirmation showed that *Cag8* was successfully disrupted in two randomly selected fluffy transformants (Fig. 3C). The complementation of *ΔCag8* was not conducted because *Cag8* had been previously characterized [17]. These data indicate the disruption of *MrKu70* dramatically increased the gene disruption efficiency in *M. robertsii*. GFP was observed in all of the 30 transformants with conidia (obtained from disruption of *Cag8* in *ΔMrKu70*), whereas GFP was not observed in all of the 281 transformants with a fluffy phenotype, showing that GFP observation excluded most transformants where the target gene was not disrupted. In addition, GFP observation could also exclude the transformants containing multiple copies of T-DNA that had inserted in the genome with one copy of T-DNA for deleting the target gene (the *egfp* cassette on the T-DNA is excluded during homologous recombination) and the other copies of T-DNA being integrated ectopically at other positions (the *egfp* cassette on the T-DNA is inserted in the genome).

## Discussion

Systematic gene disruption projects have been completed in the two ‘model’ ascomycete fungi *S. cerevisiae* (http://www-sequence.stanford.edu/group/yeast_deletion_project/deletions3.html) and *N. crassa* (http://www.dartmouth.edu/~neurosporagenome/proj_overview.html), and research based on the two projects has been of extraordinary utility in advancing our knowledge of fundamental principles of biology. However, the fungal kingdom, with an estimated 1.5 million different species [18], displays extraordinary evolutionary diversity, and model fungi such as *S. cerevisiae* and *N. crassa* cannot be used to elucidate the mechanisms involved in major fungal lifestyles that they do not share [7].

*Metarhizium robertsii* can be used as a representative to simultaneously study several major lifestyles that are not shared by the “model” fungi *S. cerevisiae* and *N. crassa*; a systematic examination of gene function in this fungus will benefit studies on other fungi. Therefore, we developed a high-throughput gene disruption methodology for *M. robertsii*, which includes two technologies. One is the modified OSCAR-based high-throughput construction of gene disruption plasmids. Using this technology, a gene disruption plasmid can be constructed in one cloning step in two days. The other technology is highly efficient gene disruption in the *MrKu70* deletion mutant. Since the disruption of *MrKu70* does not alter development, pathogenicity and tolerance to various abiotic stresses, *ΔMrKu70* can be used as a recipient strain for systematic gene disruption to characterize the full suite of genes involved in the biological processes of *M. robertsii*. However, the insect species (*G. mellonella*) used in this study is not a natural host of *M. robertsii*; therefore, so bioassays of *ΔMrKu70* should be performed when a new insect species is used. Furthermore, we propose that the wild-type strain and *ΔMrKu70* should always be used as controls to investigate the function of any gene using the gene disruption methodology. In addition, the deletion of *Ku70* genes could predispose fungal strains to DNA damaging conditions [11], [19]–[21]. Therefore, *ΔMrKu70* could been not suitable for studying genes involved in biological processes related to an intact NHEJ pathway.

In *N. crassa*, the gene deletion frequency using NHEJ-defective strains increases by increasing the size of flanking regions in the gene replacement cassette, and the gene deletion frequency reached 100% when the size was 1,000 bp. Therefore, approximately 1,000 bp of flanking regions were first used to construct the *Cag8* disruption cassette, and the gene disruption frequency was 93%. Increasing the size of the flanking regions might promote gene deletion frequency in *ΔMrKu70*; however, this was not carried out because a gene disruption frequency over 90% with an efficient fungal transformation system is sufficient for high-throughput gene deletion.

The herbicide resistance genes *Bar* or *Sur* are suitable for many other fungi such as the entomopathogenic fungus *Beauveria bassiana* [22] and the plant pathogenic fungus *Magnaporthe oryzae* [23]. Therefore, the plasmids developed in this study (pA-bar-OSCAR, pA-sur-OSCAR and pPK2-OSCAR-GFP) can be directly used for high-throughput construction of gene disruption plasmids for these fungi, possibly facilitating their functional genomic analysis.

## Materials and Methods

### Fungal and bacterial strains

*Metarhizium robertsii* ARSEF2575 was cultured as previously described [17]. *E. coli* DH5α and DB101 were used for plasmid construction. *A. tumefaciens* AGL1 was used for fungal transformation.

### Constructing OSCAR plasmids for gene disruption

In order to replace the hygromycin-resistance gene cassette (HygR) in pA-Hyg-OSCAR [14] with either of the two herbicide resistance gene cassettes (*Bar* and *Sur*), pA-Hyg-OSCAR was first digested with *Pst*I and blunted with T4 DNA polymerase. The linear plasmid was then digested with *Spe*I to remove the hygromycin-resistance gene cassette. The *Bar* cassette (cut off with *Spe*I/*Eco*RV from pPK2-bar-GFP) [24] and the *Sur* cassette (cut off with *Xba*I/*Eco*RV from pPK2-SUR-GFP) [24] were ligated with the linearized pA-Hyg-OSCAR to produce pA-Bar-OSCAR and pA-Sur-OSCAR, respectively. All restriction enzymes and DNA modification enzymes used in this study were from Fermentas (Beijing, China).

The recipient binary plasmid pPK2-OSCAR-GFP was constructed by ligating pPK2-Bar-GFP (*Bar* cassette deleted) with the DNA fragment containing a *ccdB* cassette and Bp Clonase recognition sites (attp3 and attp2r). To delete the *Bar* cassette in pPK2-Bar-GFP, the binary plasmid was digested with *Eco*RI/*Eco*RV and blunted with T4 DNA polymerase. The DNA fragment containing the *ccdB* cassette and Bp Clonase recognition sites was removed from p-OSCAR [14] with *Kpn*I/*Hind*III and blunted with T4 DNA polymerase.

### Gene disruption

The gene disruption plasmids were constructed as described by Paz et al. [14]. For each gene, two pairs of primers were designed to amplify approximately 1 Kb of the 5′ and 3′ flanking sequences, respectively, and each primer had a unique attB sequence at its 5′end. The details of all primers used in this study are described in Table 1. The PCR products, the donor plasmid (p-Bar-OSCAR or p-Sur-OSCAR), the recipient plasmid (pPK2-OSCAR-GFP) and BP Clonase (Life Technologies, Shanghai China) were prepared as described by Paz et al. [14]. After a 16-h incubation at 25°C, the mixture was transformed into *E. coli* (DH5α) mediated by CaCl_2_ [25]. Confirmation of construction of gene disruption plasmids is described in Figure 1.

**Table 1:** Primers used in this study.

Primers	Sequence (5′–3′)	Usage
Bar5	CGCCTGGACGACTAAACC	Confirmation of gene disruption
Bar3	TCAGCCTGCCGGTACCGC	
Sur5	ATCGTGGAGTCATGTTTG	Confirmation of gene disruption
Sur3	CCAGTAAGTAATATATCC	
DMrKu70-51(B2r+5F)	ggggacagctttcttgtacaaagtggaaAGCCAGGTCCCTTATCCC	Disruption of *MrKu70*
DMrKu70-52(B1r+5R)	ggggactgcttttttgtacaaacttgtCCGAGTAGAAACAATTC	
DMrKu70-31(B4+3F)	ggggacaactttgtatagaaaagttgttGACTGCTTTCATTGGTG	
DMrKu70-32(B3+3R)	ggggacaactttgtataataaagttgtTGTGCCAATTTGGCAGCC	
MrKu70-CF1	GAATAGAGCAATGGATAG	Confirmation of disruption of *MrKu70*
MrKu70-CF2	CCCTTTAGACTCGCCATC	
MrKu70-5	CCCGGGGCGTACAAGTGATGCTAC	Complementation of *ΔMrKu70*
MrKu70-3	CCCGGGAAGTGACGATTCAGATCC	
MrKu70ORF-5	ATGGGGATCAAGAGATTG	Complementation of *ΔMrKu70*
MrKu70ORF-3	GATACACCACGCAATGCC	
DCag8-51(B2r+5F)	ggggacagctttcttgtacaaagtggaaAGCAATTGTATTTGCTGG	Disruption of *Cag8*
DCag8-52(B1r+5R)	ggggactgcttttttgtacaaacttgt ACGGAGTCAAGTATGGAG	
DCag8-31(B4+3F)	ggggacaactttgtatagaaaagttgttGGTAAATACCAGCAAGTG	
DCag8-32(B3+3R)	ggggacaactttgtataataaagttgtTTTCTTATTTGCGGTTGG	
Cag8-CF1	TGTTACCACGACGACAAC	Confirmation of disruption of *Cag8*
Cag8-CF2	TTTACTGACGTGACGGTG	

Note:

B2r+5F: the Bp Clonases recognition site B2r (lowercase) and the forward primer 5F (upper case) to amplify the 5′ flanking sequences.

B1r+5R: the Bp Clonases recognition site B1r (lowercase) and the reverse primer 5R (upper case) to amplify the 5′ flanking sequences.

B4+3F: the Bp Clonases recognition site B4 (lowercase) and the forward primer 3F (upper case) to amplify the 3′ flanking sequences.

B3+3R: the Bp Clonases recognition site B3 (lowercase) and the reverse primer 3R (upper case) to amplify the 3′ flanking sequences.

To complement *ΔMrKu70*, a genomic DNA fragment of *MrKu70* including the promoter region, ORF and termination region was cloned by PCR using primers MrKu70-5 and MrKu70-3 (Table 1); this construct was then inserted into pPK2-Bar-GFP [14] to form pPK2-bar-GFP-MrKu70, which was transformed into *ΔMrKu70*.

### Testing conidial tolerance to abiotic stresses

Tolerance to cold stress (15°C), heat shock (45°C for 2 h), oxidative stress (0.005% H_2_O_2_) and high osmolarity stress (1.5 M KCl) was tested as previously described [26]. Briefly, tolerance to cold and heat shock stress was shown by the germination rate of conidia (1.5×10^6^) in a Petri dish (diameter  = 3cm) containing 2 ml of 0.01% yeast extract (Oxoid Ltd. Hampshire, England). Tolerance to heat shock was investigated by incubating conidia at 45°C for 2 h and then transferring to 26°C for continued growth. Germination was checked every 2 h. Cold stress was induced by incubating the plates at 15°C, and germination was checked at 2-h intervals. Tolerance to oxidative stress and osmolarity stress was tested by measuring the germination rate of conidia at 26°C in yeast extracts supplemented with H_2_O_2_ (0.005%) and KCl (1.5 M), respectively.

The tolerance to UV radiation was tested by measuring the germination rate of conidia treated with UV radiation [24]. Briefly, conidia were placed at a final concentration of 5×10^5^ conidia/mL in 3 ml of 0.01% yeast extract per Petri dish (diameter  = 3 cm). Petri dishes were irradiated in a UV-B GS GENE Linker UV Chamber (Bio-Rad, USA) and then either exposed to light energy (photoreactivation) for 4 h by placing the dishes 60 cm from two fluorescent light bulbs (15 W, Sylvania F15T8/CW/SS) or kept in the dark by covering immediately with aluminum foil. Conidia were then incubated at 26°C and germination was checked at 2-h intervals.

### Insect bioassay

Bioassays were conducted using last instar *Galleria mellonella* larvae (Ruiqingbait Co., Shanghai, China) as described [27]. Insects were inoculated by immersion in conidial suspensions (1×10^7^ conidia ml^−1^), and LT_50_ values were determined using the SPSS Statistical Package (SPSS Inc., Chicago, IL). All bioassays were repeated three times with 30 insects per replicate.
